# Two new species of Asellota (Crustacea, Isopoda) from coral reefs on Iriomote Island, Okinawa, Japan

**DOI:** 10.3897/zookeys.520.5943

**Published:** 2015-09-16

**Authors:** Michitaka Shimomura, Tohru Naruse

**Affiliations:** 1 Kitakyushu Museum of Natural History and Human History, Kitakyushu 805-0071, Kitakyushu, Japan; 2 Tropical Biosphere Research Center, Iriomote Station, University of the Ryukyus, 870, Uehara, Taketomi, Okinawa 907-1541, Japan

**Keywords:** Isopoda, Pleurocopidae, *Pleurocope*, Santiidae, *Prethura*, Japan

## Abstract

*Pleurocope iriomotensis* **sp. n.** and *Prethura tuberculata* **sp. n.** are described from Iriomote Island, Ryukyu Archipelago, southern Japan. These are the first records of *Pleurocope* from the Pacific and of *Prethura* from the Asian Pacific coast. *Pleurocope iriomotensis* differs from its congeners in having lateral spine-like processes on pereonite 4 and coxal plates of pereonite 7. *Prethura tuberculata* can be distinguished from its single congener in having a lateral short projection of protopod of pleopod 2.

## Introduction

**Figure 1.**
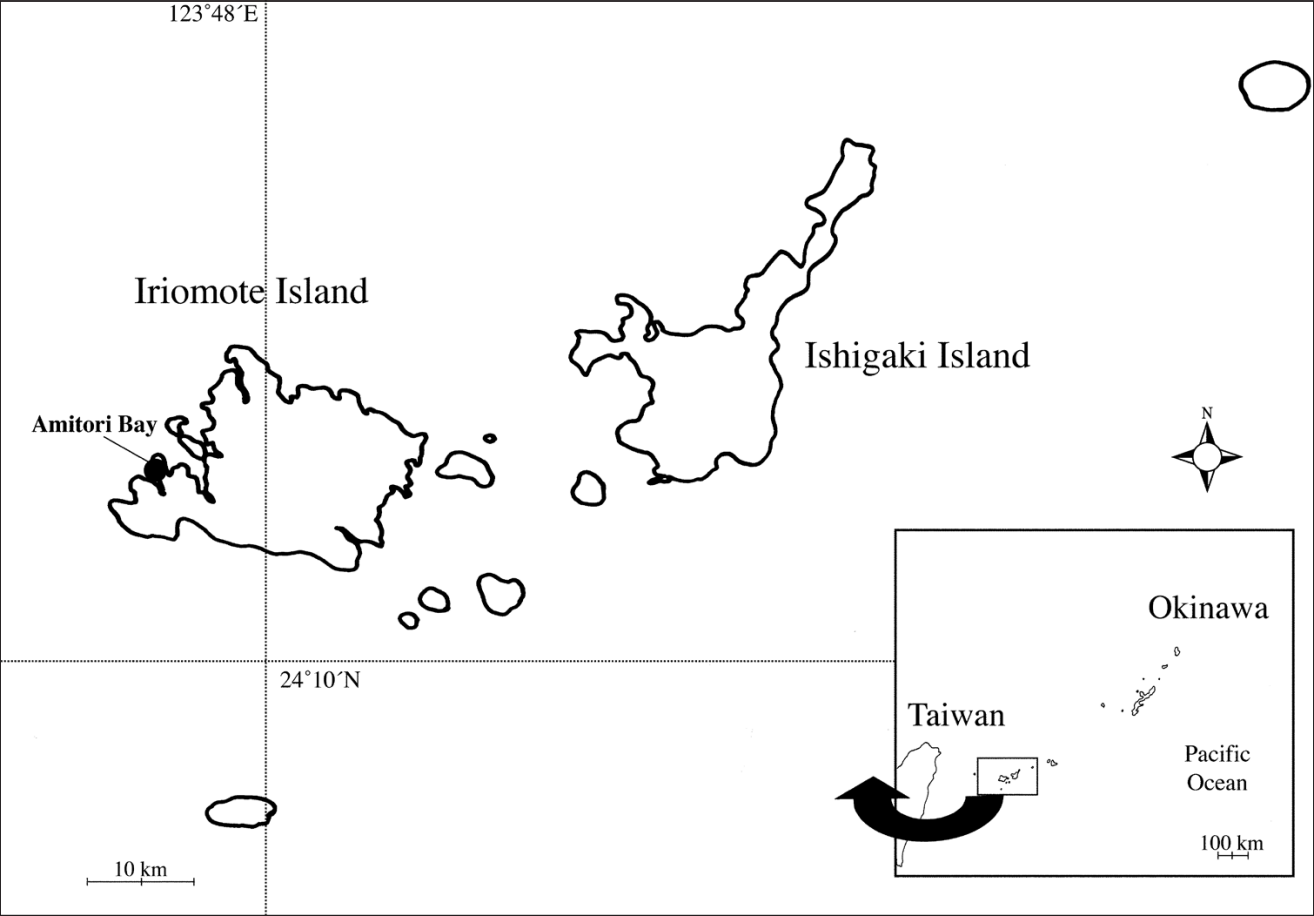
Map showing collection site (solid circle).

Iriomote Island (Fig. 1) in the southwestern part of the Ryukyu Archipelago is located in a subtropical climate zone for terrestrial environments, but its marine fauna is more tropical, being strongly influenced by Kuroshio, a prominent warm ocean current. The island is surrounded by coral reefs and a variety of mangroves, sandy or rocky shores.

The marine isopod fauna of the Ryukyus has been studied by several authors including the families Anthuridae Leach, 1814, Bopyridae Rafinesque, 1815, Cymothoidae Leach, 1818, and Gnathiidae Leach, 1814 (e.g., Nunomura 1992; Ota 2012; Shiino 1939; Williams and Williams 1986). With regards to marine asellote isopods around the Ryukyus, only the deep water species *Mastigoniscus microcephalus* (Gamô, 1989), *Munnopsis megacephalus* Shimomura & Ohtsuka, 2005 and *Heterosignum elegans* Shimomura & Mawatari, 2002 have been recorded (Gamô 1989; Shimomura and Ohtsuka 2005). The shallow-water asellote isopods have remained uninvestigated; hence we have been participating in the faunal survey of the shallow-water asellote isopods of Iriomote Island.

The shallow-water crustacean faunal survey of Amitori Bay, Iriomote Island in 2012 by SCUBA, yielded two species of asellote isopods of the families Pleurocopidae and Santiidae. Based on this material, two new species, *Pleurocope iriomotensis* sp. n. and *Prethura tuberculata* sp. n. are described and illustrated.

## Material and methods

Collections of isopods were obtained by the Collaborative Research Program funded by the Ryukyu University in Iriomote Island in 2012. Pieces of coral rubble collected by hand near the coral reefs during SCUBA were washed in a bucket, and isopods were extracted by decanting the suspension through a sieve with a mesh size of 0.3 mm. All specimens obtained were fixed and preserved in 80% ethanol. Each individual was dissected and prepared for observation by a light microscope (Nikon E600). The total length as indicated in “Material examined” was measured from the tip of the head to the end of the pleotelson.

The type specimens are deposited in the Kitakyushu Museum of Natural History and Human History (KMNH).

## Systematics

### Family Pleurocopidae Fresi & Schiecke, 1972

#### Pleurocope

Taxon classificationAnimaliaIsopodaPleurocopidae

Walker, 1901

Pleurocope Walker, 1901: 297; Kensley and Schotte 2002: 1435.

##### Type species.

*Pleurocope dasyura* Walker, 1901 (by original designation and monotypy).

##### Species included.

*Pleurocope dasyura* Walker, 1901, Mediterranean; *Pleurocope floridensis* Hooker, 1985, Gulf of Mexico; *Pleurocope wilsoni* Kensley & Schotte, 2002, Thailand and Seychelles; *Pleurocope iriomotensis* **sp. n.**, Iriomote Island, Ryukyu Islands (present study).

##### Diagnosis

**(modified from Kensley and Schotte 2002).** Antennular flagellum composed of 4 articles. Antennal flagellum composed of 6–7 articles. Mandible without palp. Maxillula medial lobe rudimentary, without setae; lateral lobe with 7–9 setae apically. Maxilla lateral 2 lobes each with 2 setae apically. Some pereonites with spine-like process laterally, often bearing 1–2 stout setae. Pereopod 1 subchelate. Pereopods 2–7 with single claw. Coxal plates of pereopods 5–7 visible in dorsal view. Some coxal plates with spine-like process laterally, with 1–3 stout setae. Uropods composed of protopod, endopod and exopod, inserted dorsolaterally on proximal pleon. Pleotelson tapering posteriorly to acute apex. Male pleopod 1 tapering posteriorly, with some short setae on apex. Male pleopod 2: protopod narrow, lanceolate, with single long seta on apex; second article reaching or surpassing apex of protopod.

##### Remarks.

Walker (1901) established the genus for his new species *Pleurocope dasyura* from a depth of 18 m at the Mediterranean. A second species, *Pleurocope floridensis*, was described by Hooker (1985) from a depth of 30 m at the Florida Middlegrounds in the northeastern Gulf of Mexico. Kensley and Schotte (2002) redefined the genus with the description of the new species of *Pleurocope wilsoni* from a depth of 77 m off Phuket Island, Thailand, and a depth of 6–16 m at Picard Island, Aldabra Atoll.

The main changes in this new diagnosis are to accommodate the number of seta on maxillae.

#### Pleurocope iriomotensis sp. n.

Taxon classificationAnimaliaIsopodaPleurocopidae

http://zoobank.org/D687B687-10AE-491F-94CA-63C99CF6A3C3

Figs 2 , 3 , 4

##### Material examined.

Holotype. ♂ (0.9 mm), 24°20´N, 123°41´E, Amitori Bay, Okinawa, Japan, 19 July 2012, dead coral, 25 m, coll. TN (KMNH IvR 500,729).

Paratypes. 3♂♂ (0.5–0.9 mm), Amitori Bay, Okinawa, Japan, 19 July 2012, dead coral, 4 m, coll. MS (KMNH IvR 500,730–500,732).

##### Description of the holotype.

**Figure 2.**
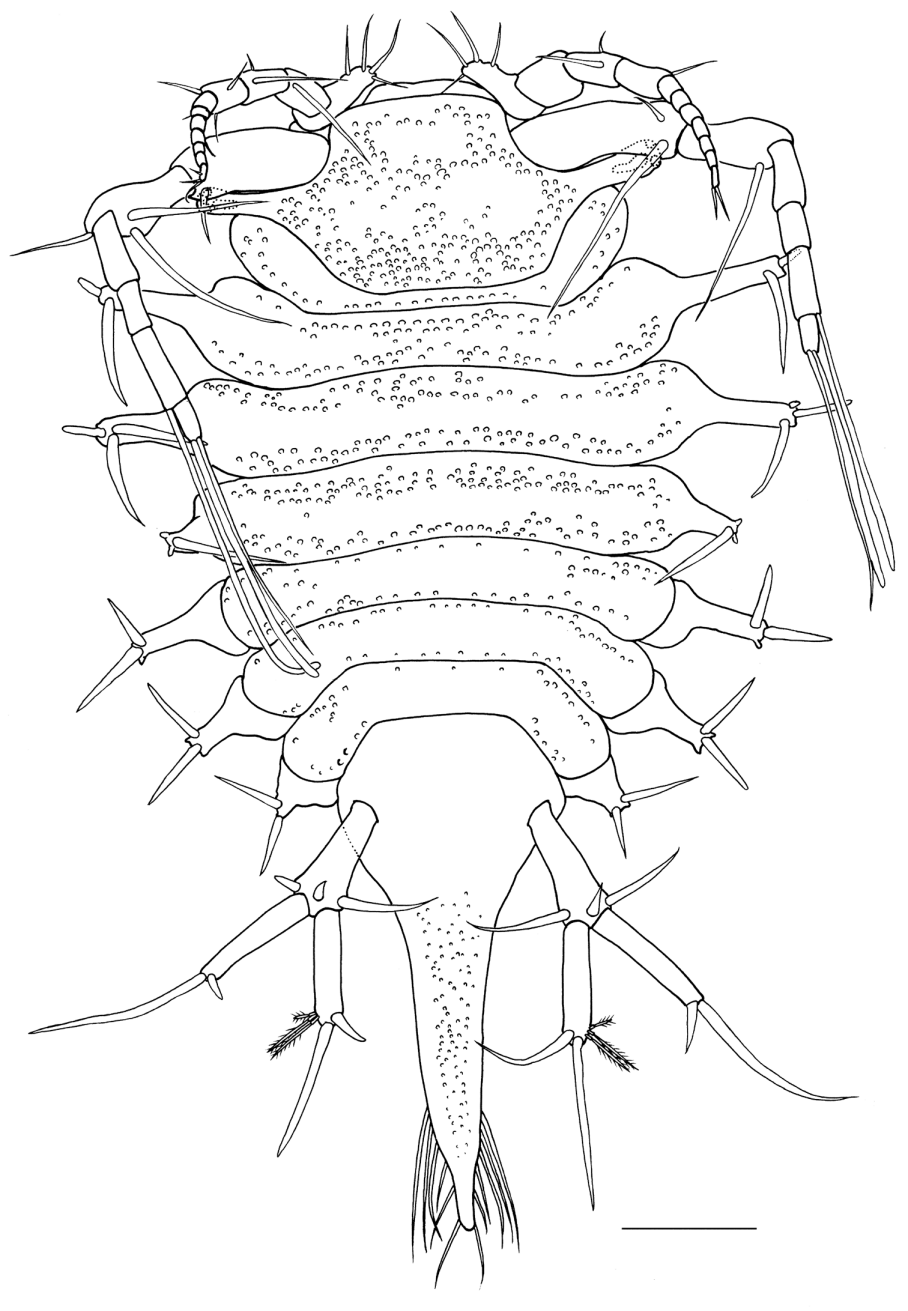
*Pleurocope iriomotensis* sp. n., holotype male, habitus, dorsal. Scale bar: 100 μm.

*Body* (Fig. 2) 1.6 times as long as maximum width (including spine-like processes), widest at pereonite 3, with many small granules on dorsum, without dorsal setae. Head (including eyestalks) 2.4 times as broad as long, head without eyestalks 1.2 times as broad as long, broader than pereonite 1; frontal and posterior margins of head convex. Eyes each with 2 ommatidia; eyestalks slender, long, reaching near distal end of article 1 of antenna 1. Pereonites 1 and 5–7 laterally rounded, without lateral spine-like processes; pereonites 2 and 3 with pair of long lateral spine-like processes bearing 2 or 3 robust setae; lateral spine-like processes of pereonites 2 and 3 reaching to tip of second article of antennula in length; pereonite 4 with pair of short lateral spine-like processes bearing single robust seta and 2 short teeth distally; lateral spine-like processes of pereonite 4 half as long as ones of pereonite 3. Pereonite 1 shortest; pereonite 2 three times as long as pereonite 1; pereonites 3 and 4 longest, subequal in length; pereonite 5 0.7 times as long as pereonite 4, pereonites 5–7 subequal in length. Pereonites 1 to 3 increasing in width; pereonite 4 narrower than pereonites 3; pereonites 4 to 7 decreasing in width. Coxal plates dorsally visible on pereonites 5–7, laterally with spine-like process and 2 robust setae. Pleotelson (Fig. 2) approx. 2.2 times as long as wide, widest at anterior one seventh, tapering to sharply rounded apex, with 12 robust setae distally.

**Figure 3.**
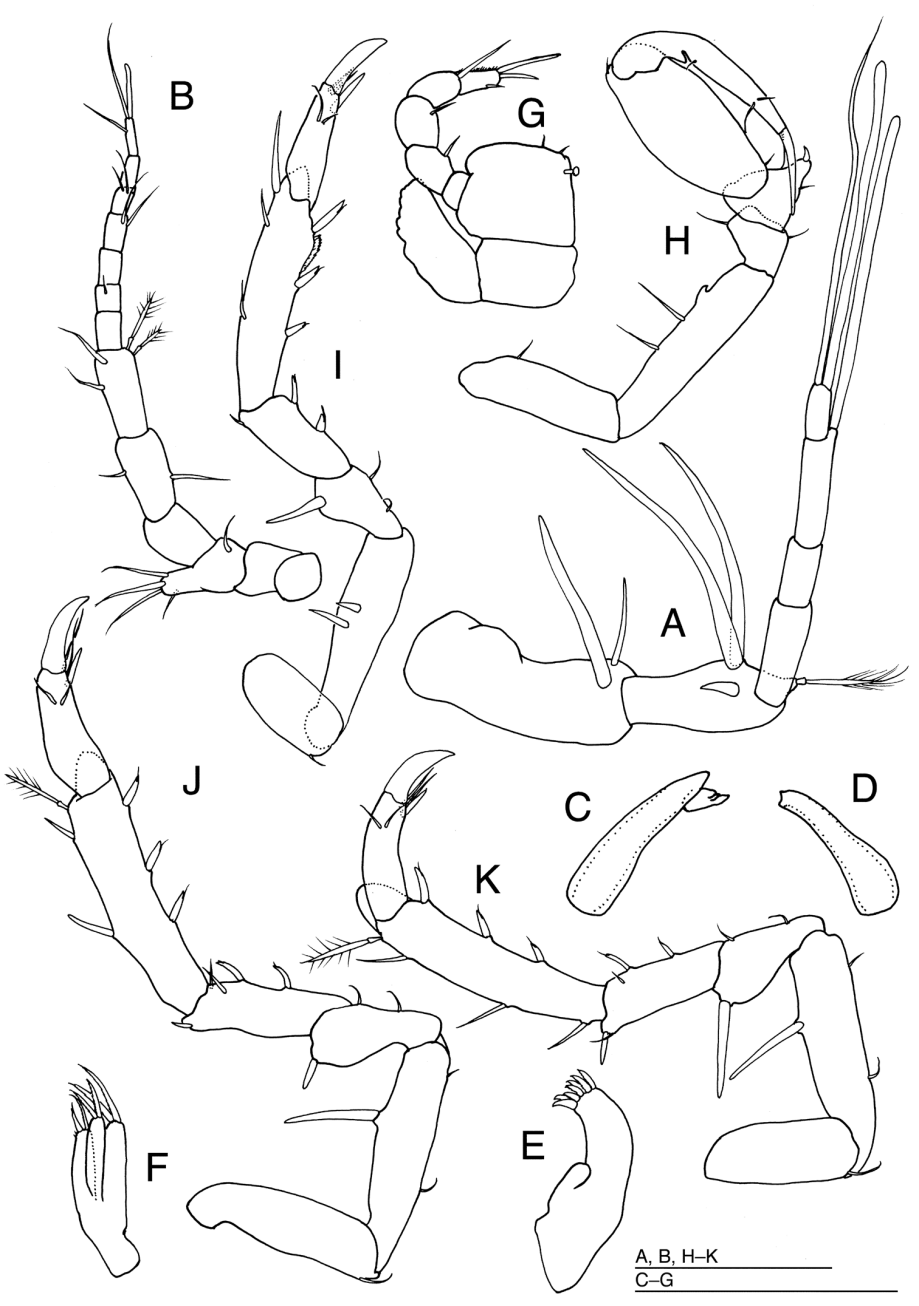
*Pleurocope iriomotensis* sp. n. **A–B, E–K** holotype male **C–D** paratype male (KMNH IvR 500,732): **A** left antennula, dorsal **B** left antenna, ventral **C** left mandible, dorsal **D** right mandible, dorsal **E** left maxillula, ventral **F** left maxilla, ventral **G** left maxilliped, dorsal **H** left pereopod 1, medial; **I** left pereopod 2, medial **J** left pereopod 3, medial **K** left pereopod 4, medial. Scale bars: 100 μm.

*Antennula* (Fig. 3A) consisting of 6 articles. Article 1 longest and broadest, twice broader than eyestalks, with 1 long and 1 short simple setae laterodistally; article 2 nearly 0.8 times as long as article 1, distally with 1 short and 2 long, stout simple setae and 1 broom-like seta; article 3 about half as long as article 2, without setae; article 4 approx. 0.6 times as long as article 3, without setae; article 5 approx. 1.8 times as long as article 4, with 1 aesthetasc distally; article 6 shortest, with 1 long simple seta and 1 aesthetasc apically.

*Antenna* (Fig. 3B): peduncle consisting of 4 short and 2 long articles, and flagellum of 7 short articles. Articles 1 and 2 subquadrate, without setae; article 3 with a simple seta laterodistally and 4 simple setae on lateral protrusion; article 4 as long as articles 1 and 2 combined, without setae; article 5 longer than article 4, with single simple seta medially and laterally; article 6 as long as article 5, with 2 simple setae distomedially and 2 broom-like setae laterodistally; flagellum as long as peduncular articles 4–6 together, flagellar articles 1–6 each with 0, 1, 0, 2, 2 and 0 simple setae distally; flagellar article 7 with 2 simple setae and 1 aesthetasc.

*Maxillula* (Fig. 3E) with medial lobe short, lacking setae; lateral lobe with 7 setae distally. *Maxilla* (Fig. 3F) with medial lobe bearing some fine and 2 stout setae on margin; lateral 2 lobes each with 2 stout setae apically.

*Maxilliped* (Fig. 3G) palp slender, twice as long as basis: article 1 shortest, without setae; articles 2 to 4 subequal in length, each with single distal seta; article 5 narrowest, with a short seta medially and 2 stout setae apically; basis quadrate with broad endite bearing 2 simple setae distally and 1 coupling hook medially; epipod crenulated laterally, 2.6 times as long as width, 1.5 times as long as basis.

*Pereopod 1* (Fig. 3H): basis 0.8 as long as ischium, with 1 dorsal seta; ischium the longest article, with short tooth and 2 long setae dorsally; merus pentagonal, with a ventral and a dorsal seta; carpus trapezoidal, as long as merus, with distoventral tapering projection terminating with 1 stout seta and 1 slender setae ventrally; propodus ovate, 2.3 times as long as width, with 1 proximoventral and 1 distodorsal small setae; dactylus as long as propodus, with 1 triangulate tooth and 1 short seta ventrally and 2 setae subapically, unguis half as long as dactylus and 1 slender seta apically.

**Figure 4.**
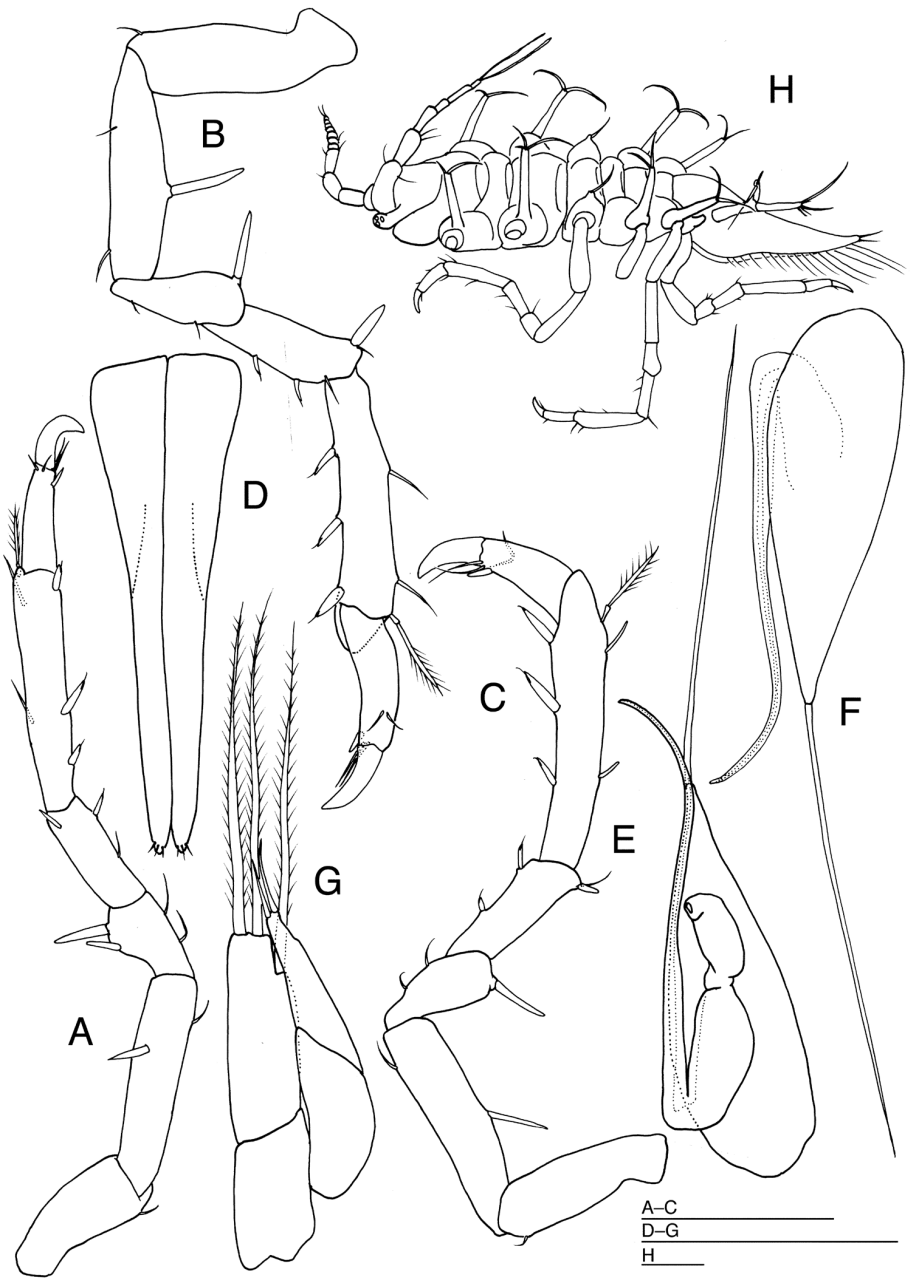
*Pleurocope iriomotensis* sp. n. **A–G** holotype male **H** paratype male (KMNH IvR 500,731): **A** left pereopod 5, medial **B** left pereopod 6, medial **C** left pereopod 7, lateral **D** pleopod 1, ventral **E** left pleopod 2, dorsal **F** left pleopod 2, ventral **G** left pleopod 3, dorsal **H** habitus, lateral. Scale bars: 100 μm.

*Pereopod 2* (Fig. 3I) shorter than pereopods 3–7; basis with 1 distoventral seta; ischium 2.1 times as long as basis, dorsally with 2 robust setae; merus shorter than half length of ischium, with 2 simple setae ventrally and 1 robust seta dorsally; carpus longer than merus, with 2 robust setae ventrally and 1 simple seta dorsodistally; propodus as long as ischium, ventrally with 3 robust unequally bifid setae and 1 fringed scale on distal third, and with 2 simple and 1 robust seta dorsally; dactylus slender, 3.1 times as long as width, with 1 simple seta, 1 stout unguis and 1 accessory claw apically, and with 2 simple setae subapically. *Pereopods 3–7* (Figs 3J–K, 4A–C) subequal in shape and slightly increasing in length posteriorly; bases with 1 ventrodistal seta; ischia longer than bases, with 1–2 simple ventrally and 1–2 robust setae dorsally; meri less shorter than half length of ischia, with 2 setae ventrally and 1–2 robust setae dorsally; carpi longer than meri, with 1–2 simple and 2 robust setae ventrally and 0–1 simple and 0–1 robust setae dorsally; propodi longer than ischia, with 3 robust setae ventrally and 2–3 robust and 1 broom-like seta dorsally; dactyli with 2 simple setae, 1 stout unguis and 1 short accessory claw apically and 2 simple setae subapically.

*Pleopod 1* (Fig. 4D) about 3.4 times as long as maximum width, tapering to apex, apically with 3 pairs of short setae. *Pleopod 2* (Fig. 4E, F): protopod 3 times as long as wide, bearing long seta apically; apical seta 1.2 times as long as protopod; endopod surpassing tip of protopod, with slender, long second article; second article 1.2 times as long as protopod; exopod narrower than endopod first article. *Pleopod 3* (Fig. 4G): endopod 4.7 times as long as width, bearing 3 stout plumose setae distally; exopod 0.9 times as long as endopod, bearing 2 simple setae apically.

*Uropod* (Fig. 2) half as long as pleotelson. Protopod wide posteriorly, with 3 robust setae distally; exopod as long as protopod, with 2 robust setae distally; endopod slightly shorter than exopod, with 3 broom-like and 2 robust setae distally.

##### Description of the paratypes.

*Body* (Fig. 4H) flattened, without spines and projections on dorsum. *Left mandible* (Fig. 3C) incisor 4 conical, directed anteriorly, without setae and teeth; lacinia mobilis with 3 teeth. *Right mandible* (Fig. 3D) incisor with 2 teeth apically, without setae.

##### Female.

Not known.

##### Remarks.

*Pleurocope iriomotensis* sp. n. can be identified by the following combination of characters: pereonite 1 lacking lateral spine-like processes; pereonites 2–4 and coxal plates of pereonites 5–7 each with lateral spine-like process; pleotelson twice as long longer as wide; flagellum of antenna consisting of 7 articles. Pereonite 1 without a spine-like process links the new species to *Pleurocope floridensis* Hooker, 1985, from the Gulf of Mexico and to *Pleurocope dasyura* Walker, 1901, from the Gulf of Naples, Italy. *Pleurocope iriomotensis* is distinguished from *Pleurocope floridensis* by the following characters (those of *Pleurocope floridensis* in parentheses): pereonite 4 and coxal plate of pereonite 7 with spine-like process (without spine-like process); eyestalks not reaching to second article of antennula (surpassing second article of antennula); fifth article of antennula with 1 aesthetasc (with 2 aesthetascs); ischium of pereopod 1 with dorsal projection (without dorsal projection); pleopod 1 apically with 3 pairs of short setae (apically with 5 pairs of short setae). *Pleurocope dasyura* differs from the new species in having pereonite 4 and coxal plate of pereonite 7 without spine-like processes, pereon with 6 long dorsal setae, antenna with long projection, and pleopod 1 apically with 4 pairs of short setae.

##### Etymology.

The species is named after the type locality.

### Key to the species of *Pleurocope*

**Table:** 

1	Lateral spine-like process on pereonite 4 and coxal plate of pereonite 7 absent	**2**
–	Lateral spine-like process on pereonite 4 and coxal plate of pereonite 7 present	***Pleurocope iriomotensis* sp. n.**
2	Lateral spine-like process on pereonite 1 absent; pleotelson twice longer than width	**3**
–	Lateral spine-like process on pereonite 1 present; pleotelson twice as long as width	***Pleurocope wilsoni***
3	Lateral seta on pereonite 4 absent; dorsal short tooth of ischium of pereopod 1 absent	***Pleurocope floridensis***
–	Lateral seta on pereonite 4 present; dorsal short tooth of ischium of pereopod 1 present	***Pleurocope dasyura***

### Family Santiidae Wilson, 1987

#### Prethura

Taxon classificationAnimaliaIsopodaSantiidae

Kensley, 1982

Prethura Kensley, 1982: 255; Wolff 1989: 181.

##### Type species.

*Prethura hutchingsae* Kensley, 1982; by original designation and monotypy.

##### Species included

*Prethura hutchingsae* Kensley, 1982, Great Barrier Reef, Australia; *Prethura tuberculata* sp. n., Iriomote Island, Ryukyu Islands (present study).

##### Diagnosis

**(modified from Wolff 1989).** Pleotelson subtriangular. Eyes well-developed, on short peduncles. Mandibular palp lacking. Coxal plates of pereonites 5 visible dorsally. Male pleopod 1 distally twisted, apically acute. Male pleopod 2 protopod enormously expanded. Uropods pedunculate, protopod enlarged, inserted ventrolaterally; medial ramus short. Female operculum pyriform.

##### Remarks.

The generic diagnosis is slightly modified from that of Wolff (1989). The significant change in this new diagnosis is to accommodate the presence of well-developed eyes on short peduncles.

#### Prethura tuberculata sp. n.

Taxon classificationAnimaliaIsopodaSantiidae

http://zoobank.org/D79017CF-FDB4-4DB2-8643-968E2EA7E87E

Figs 5 , 6

##### Material examined.

Holotype. ♂ (0.9 mm), 24°20´N, 123°41´E, Amitori Bay, Okinawa, Japan, 19 July 2012, dead coral, 4 m, coll. MS (KMNH IvR 500,733).

##### Description of the holotype.

**Figure 5.**
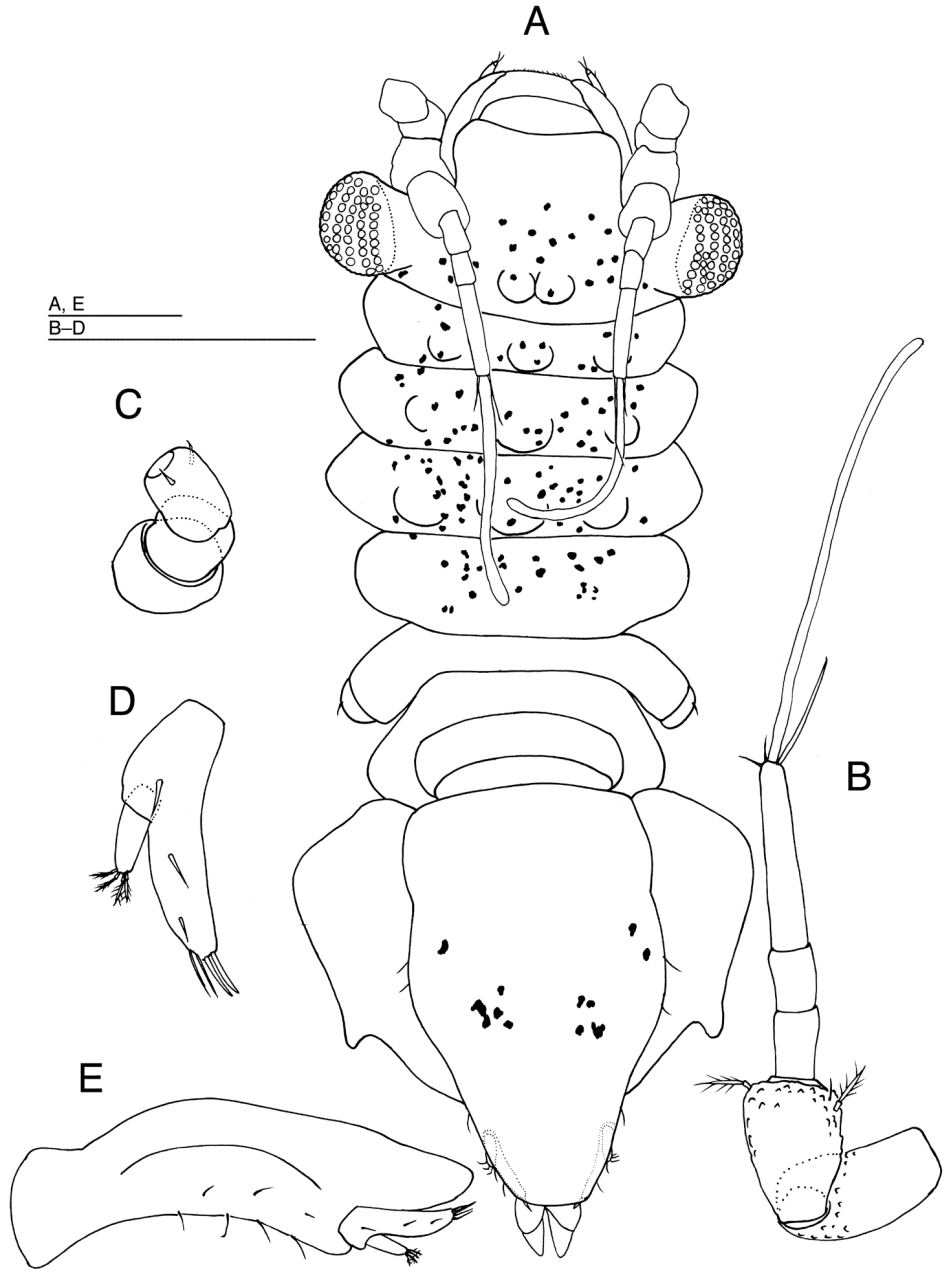
*Prethura tuberculata* sp. n. **A–E** holotype male: **A** habitus, dorsal **B** right antennula, dorsal **C** articles 1–3 of antenna 2, dorsal **D** left uropod, lateral **E** pleotelson and uropod, lateral. Scales = 100 μm.

*Body* (Fig. 5A) 2.6 times as long as maximum width, widest at head, with many small black chromatophores. Head 2.3 times as broad as long, broader than pereon, with pair of rounded protuberances dorsally; frontal margin concave; posterior margin slightly convex. Eyes each with 43 ommatidia. Pereonites 1 to 3 increasing in length posteriorly, each with 3 rounded protuberances dorsally; pereonite 4 longer than pereonite 3, without protuberances; pereonite 5 shortest; pereonite 6 slightly longer than pereonite 5; pereonite 7 as long as pereonite 6. Pereonites 1 to 3 increasing in width; pereonite 4 narrower than pereonite 3; pereonite 5 wider than pereonite 4; pereonite 6 narrower than pereonite 5; pereonite 7 narrowest. Pleonite half as long as pereonite 7, without pigmentations and setae dorsally. Pleotelson (Fig. 5A, E) 1.7 times as long as wide, with 3 pairs of short setae laterally and 4 pairs of short setae marginally.

*Antennula* (Fig. 5B) consisting of 5 articles. Article 1 with many granules, without setae; article 2 broadest, as long as article 1, with many granules, and with 1 simple seta and 1 broom-like seta distomedially and 1 broom-like seta distolaterally; article 3 and 4 subequal in length, without setae; article 5 longest, approx. 3.3 times as long as article 4, with 3 simple setae and 1 aesthetasc apically.

*Antenna* (Fig. 5C): articles 1 and 2 without setae; article 3 narrower than article 2, with 2 short setae distally; articles 4–6 and flagellar articles broken.

**Figure 6.**
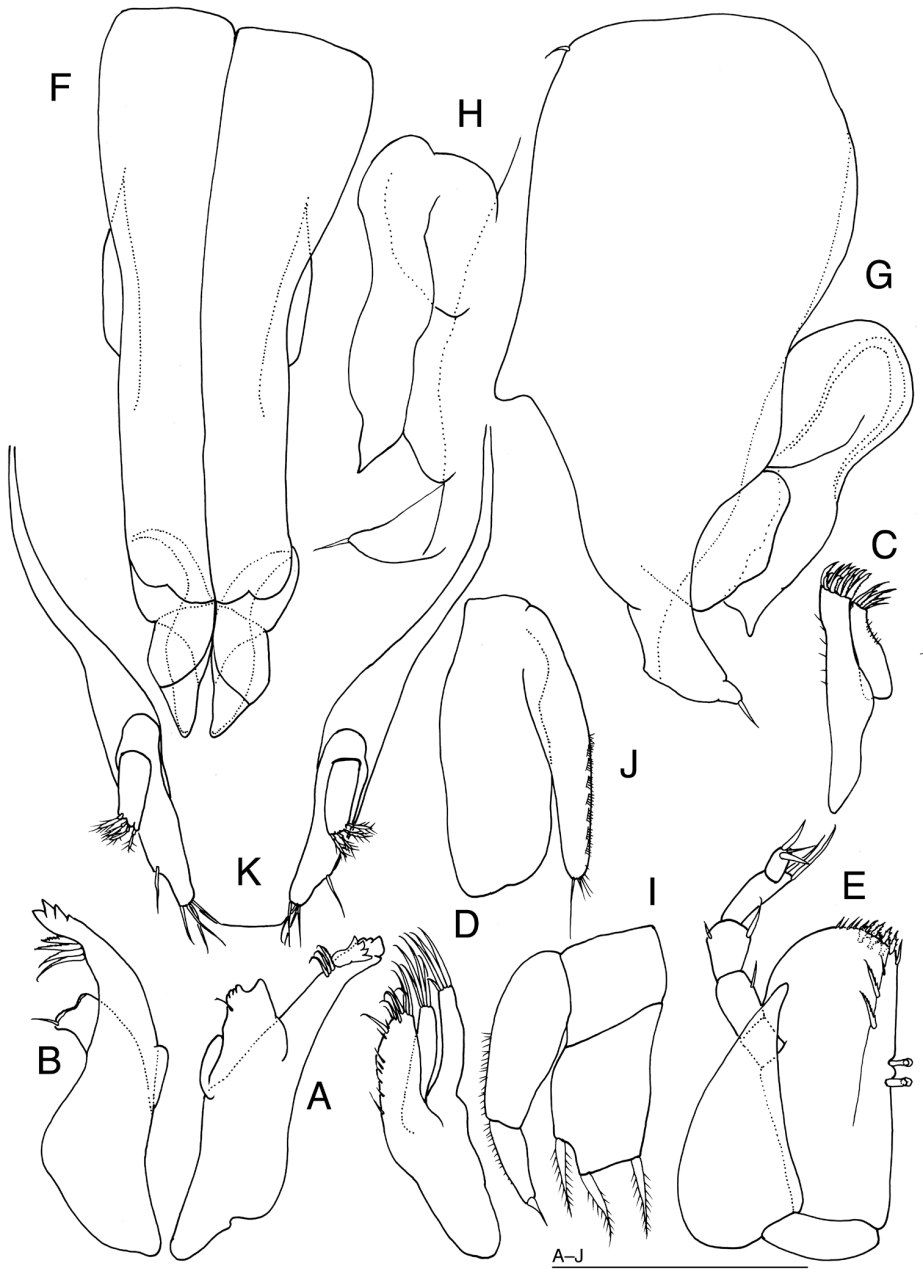
*Prethura tuberculata* sp. n. **A–K** holotype male: **A** left mandible, medial **B** right mandible, dorsal **C** left maxillula, dorsal **D** left maxilla, ventral **E** left maxilliped, dorsal **F** pleopod 1, ventral **G** right pleopod 2, ventral **H** endopod of right pleopod, dorsal **I** left pleopod 3, dorsal **J** left pleopod 4, ventral **K** pleotelson and uropods, ventral. Scale bar: 100 μm.

*Left mandible* (Fig. 6A) incisor with 4 teeth, 4-toothed lacinia mobilis and 3 serrated setae; molar process cylindrical, with one large and four short teeth, and 1 seta distally. *Right mandible* (Fig. 6B) incisor with 4 teeth and 4 serrate setae; molar process cylindrical, with 1 seta distally.

*Maxillula* (Fig. 6C): medial lobe with 5 setae apically and some fine setae medially; lateral lobe with 12 setae apically and some fine setae laterally. *Maxilla* (Fig. 6D) with medial lobe bearing some fine and 7 stout setae medially; two lateral lobes each with 4 stout setae apically.

*Maxilliped* (Fig. 6E) palp slender, approx. 0.8 times as long as basis: article 1 without setae; articles 2 about twice as long as article 1, with 1 medial seta; article 3 longer than half of article 2, with 1 seta medially and laterally; article 4 longest, with small distomedial lobe bearing 2 long setae; article 5 shortest, with 3 apical setae. Basis quadrate, bearing one simple and two pectinate setae on dorsal ridge, some simple and irregular shaped setae distally, 3 fan-shaped setae submarginally, and 2 coupling hooks medially. Epipod lanceolate, with acute apex. Pereopods broken.

*Pleopod 1* (Fig. 6F) about 2.5 times as long as maximum width, without setae. *Pleopod 2* (Fig. 6G, H) with broad protopod bearing simple seta apically; protopod with short projection and simple seta laterally; endopod broad, with stout, short stylet; exopod stout. *Pleopod 3* (Fig. 6I) protopod as long as width, shorter than endopod; endopod 1.6 times as long as width, with 3 short, plumose setae distally; exopod 0.8 times as broad as endopod, bearing 1 simple seta apically and many fine setae laterally. *Pleopod 4* (Fig. 6J) endopod ovate, 2.7 times as long as broad; exopod its tip not surpassing tip of endopod, distally with 1 long slender and some short setae, and laterally with 7 spinulose scales. *Pleopod 5* broken.

*Uropod* (Figs 5D, 6K) 0.2 times as long as pleotelson. Protopod widest at anterior part, narrow posteriorly, with 4 simple setae distally and 3 simple setae laterally; endopod 0.3 times as long as protopod, cylindrical, with 4 broom-like setae apically.

##### Remarks.

This new species differs from the only congener, *Prethura hutchingsae* Kensley, 1982, from the Great Barrier Reef, by the following characters (those of *Prethura hutchingsae* in parentheses): pleopod 1 lacking any setae (with setae); protopod of pleopod 2 with lateral projection (without projection); epipod of maxilliped with acute apex (blunt apex).

##### Etymology.

From the Latin “*tuberculatus*”, referring to the lateral projection of the protopod of pleopod 1.

## Supplementary Material

XML Treatment for Pleurocope

XML Treatment for Pleurocope iriomotensis

XML Treatment for Prethura

XML Treatment for Prethura tuberculata
